# Computational Methods and Models in Circulatory and Reproductive Systems

**DOI:** 10.1155/2016/9028409

**Published:** 2016-11-24

**Authors:** Fang-Bao Tian, Yi Sui, Luoding Zhu, Chang Shu, Hyung J. Sung

**Affiliations:** ^1^School of Engineering and Information Technology, University of New South Wales, Canberra, ACT 2600, Australia; ^2^School of Engineering and Materials Science, Queen Mary University of London, London E1 4NS, UK; ^3^Department of Mathematical Sciences, Indiana University-Purdue University Indianapolis, 402 North Blackford Street, Indianapolis, Indiana 46202, USA; ^4^Department of Mechanical Engineering, National University of Singapore, 10 Kent Ridge Crescent, Singapore 119260; ^5^Department of Mechanical Engineering, Korea Advanced Institute of Science and Technology (KAIST), 373-1 Guseong-dong, Yuseong-gu, Daejeon 305-701, Republic of Korea

## 1. Introduction

The circulatory and reproductive systems can be considered as tubular transport systems of blood cells and gametes, respectively. Both systems involve complex flows and fluid-structure interactions of large displacements and large deformations. In addition, multiple scales and multiple processes need to be considered. Because of their importance in health and fundamentals, great effort has been put in developing numerical methods for these systems and in understanding the underlying flow physics. In spite of the many achievements, it is still a challenge to model these systems accurately using numerical methods. Moreover, there still remains much concerted development towards grasping the complicated flow physics within such systems by using advanced numerical methods and mathematical models. Therefore, the goal of this special issue was to collect together recent contributions on the development of numerical methods for complex flows and fluid-structure interaction in circulatory and reproductive systems and/or on addressing the complicated flow physics and heat and mass transfer in these systems for fundamental understanding as well as engineering applications. The papers can be divided into three groups as detailed below.

## 2. Patient-Specific Geometry Simulations

The work by J. S. Byun, S.-Y. Choi, and T. Seo attempts to investigate the hemodynamic phenomena in the cerebral arteries before and after surgery of the aneurysm with patient-specific pre- and postsurgery cerebral arterial geometries by using three-dimensional computational fluid dynamics models. In the models, the flow is approximated by an incompressible and Newtonian fluid in laminar flow regime, and the vascular wall is assumed to be rigid. The flow patterns, the inflow jet streams, and the wall average shear stress have been considered by using different patient-specific geometries. Several interesting findings are reported, among which the average shear stress distribution associated with aneurysm rupture is attractive and could provide useful insight in predicting the risk of aneurysm rupture.

X. Liu et al. present a numerical study of flow characteristics in the patient-specific upper airway obstructed by the pharyngeal collapse. In this study, the patient-specific geometries are used, the flow dynamics is solved by using computational fluid dynamics, and the turbulence is modeled by the large eddy simulation. The numerical simulations are validated with their measurement by a laser Doppler anemometry. Simulations are conducted by varying flux rate under both continuous inspiration and expiration with focus on discussing the velocity fields and static pressure fields and their correlation with the upper airway statuses (e.g., narrowing of pharynx). Such information could provide guidance for the treatment of obstructed respiratory disease. In addition, the numerical procedures reported can be directly applied to study flows in narrowed artery, arteriovenous graft, and arteriovenous fistula.

## 3. Cell/Capsule Dynamics

J.-T. Ma, Y.-Q. Xu, and X.-Y. Tang report a size-dependent cell sorting scheme based on a controllable asymmetric pinched flow by using an immersed boundary-lattice Boltzmann method. The scheme employs a device which consists of 2 upstream branches, 1 transitional channel, and 4 downstream branches. To study the cell sorting efficiency, systematic simulations are conducted by varying the inlet flow ratio, the cell size, and the outlet flow ratio. The device is approved to be effective by the finding that cells of different diameters can be successfully sorted into different downstream branches. This work provides a reference for the design of microfluidics for cell/particle sorting.

The work by F.-B. Tian presents an immersed boundary-lattice Boltzmann method for capsule fluid-structure interaction involving non-Newtonian fluid (e.g., power-law fluid). In this method, the capsule dynamics and the fluid dynamics are coupled by using the immersed boundary method; the incompressible viscous power-law fluid dynamics is obtained by solving the lattice Boltzmann equation where a shear rate-dependant relaxation time is used to achieve the non-Newtonian rheology. The non-Newtonian flow solver is validated by considering a power-law flow in a channel and then applied to study the deformation of a capsule in a power-law shear flow by varying the Reynolds number, dimensionless shear rate, and power-law index. Several interesting results that are distinctive in the power-law flow are reported. This work provides an alternative for fluid-structure interactions involving non-Newtonian fluid. Further effort can be made to explore applications of non-Newtonian effects in cell/particle sorting.

## 4. Molecular Dynamics Simulation

Y. Ge et al. present a study on the effects of solution concentration on ion distribution in a nanopore-based device inspired from red blood cells by using a molecular dynamics method. It is found that the density peaks for both the counterion and coion near the charged wall increase at different speeds with the increase of the solution concentration if the screening effects appeared, and consequently the potential near the charged wall of the nanopore switches from a negative value to a positive one during the simulation. This work provides insights in controlling the ion permeability and improving the cell transfection as well as the design of nanofluidic devices. In addition, it inspires future work on multiscale methods including the method in this work and those methods in the studies by F.-B. Tian and J.-T. Ma, Y.-Q. Xu, and X.-Y. Tang to tackle multiscale cell dynamics.

The wide biomedical and mechanical engineering science community will be interested in this special issue. Both researchers and practitioners will find results presented in this issue helpful in many applications.

*Fang-Bao Tian* *Fang-Bao Tian* *Yi Sui* *Yi Sui* *Luoding Zhu* *Luoding Zhu* *Chang Shu* *Chang Shu* *Hyung J. Sung* *Hyung J. Sung*

